# Interleukin-1 receptor-dependent and -independent caspase-1 activity in retinal cells mediated by receptor interacting protein 2

**DOI:** 10.3389/fcell.2024.1467799

**Published:** 2024-10-16

**Authors:** Brandon A. Coughlin, Barbara Christian, Brett Trombley, Susanne Mohr

**Affiliations:** Department of Physiology, Michigan State University, East Lansing, MI, United States

**Keywords:** diabetic retinopathy, Müller cells, retinal inflammation, caspase-1, interleukin-1beta, receptor interacting protein 2

## Abstract

**Introduction:**

Inflammation and cell death play an important role in the pathogenesis of diabetic retinopathy. Previously we observed sustained activation of pro-inflammatory caspase-1 in retinas of diabetic animals and patients. In this study, we aimed to look at mechanisms underlying chronic caspase-1 activation *in vitro* and *in vivo*.

**Methods:**

Non-diabetic and diabetic wild type and IL-1 receptor (IL-1R1) knockout mice were used for *in vivo* experiments. Diabetes was induced using STZ (streptozotocin). Human Müller cells were used for *in vitro* studies. Cells were treated with either 5 mM or 25 mM glucose or interleukin-1beta (IL-1β) in the presence or absence of IL-1 receptor antagonist (IL-1ra) or siRNA against RIP2 (receptor interacting protein-2) for up to 96 h. Outcome measurements to assess Müller cell functions included measurements of caspase-1 activity using a fluorescence peptide substrate, production of IL-1β by Elisa, and cell death using trypan blue exclusion assays.

**Results:**

Our *in vivo* results demonstrate that caspase-1 activation progresses from an IL-1R1 independent mechanism at 10 weeks of diabetes to an IL-1R1 dependent mechanism at 20 weeks indicating that feedback through IL-1R1 is crucial for sustained caspase-1 activity in retinas of mice. A similar hyperglycemia-mediated caspase-1/IL-1β/IL-1R1 feedback signaling was detected *in vitro* in human Müller cells which was prevented by treatment with IL-1ra. Our data also indicate that hyperglycemia induces caspase-1 activation initially but IL-1β sustains caspase-1 activation via caspase-1/IL-1β/IL-1R1 feedback and we identified RIP2 as mediator for both hyperglycemia- and IL-1β-induced caspase-1 activation. Activation of caspase-1/IL-1β/IL-1R1 feedback signaling caused Müller cell death which was prevented by RIP2 knockdown.

**Discussion:**

We conclude that any intervention in caspase-1/IL-1β/IL-1R1 feedback signaling presents novel therapeutic options for the treatment of diabetic retinopathy.

## 1 Introduction

Diabetes leads to many complications, one of them being diabetic retinopathy. Diabetic retinopathy is characterized by microaneurysms, vascular leakage, neovascularization and/or macular edema within the retina and ultimately leads to vision loss (Stitt et al., 2013). Diabetic retinopathy was once thought of as a purely microvascular disease, however studies now suggest that the disease originates within the retinal tissue. Sustained low-grade retinal tissue inflammation is now considered a cause for retinal cell dysfunction and cell death leading to subsequent microvascular changes (Abcouwer, 2012; Adamis, 2002; Coughlin et al., 2019a). Despite increasing evidence that chronic inflammation and cell death contribute to development and progression of diabetic retinopathy, there have been few treatments aimed at preventing these events. To develop such treatments, a better understanding of mechanisms underlying initiation and maintenance of chronic inflammation and inflammation patterns is crucial.

Interleukin-1β (IL-1β) has emerged as one of the most prominent pro-inflammatory cytokines associated with diabetic retinopathy (Yoshida et al., 2004; Sfikakis et al., 2005; Demircan et al., 2006; Joussen et al., 2009; Li et al., 2009; Garber and Zhu, 2023). Levels of IL-1β are elevated in the vitreous and serum of patients with proliferative diabetic retinopathy compared to healthy individuals (Demircan et al., 2006). Caspase-1, originally named Interleukin-1β Converting Enzyme (ICE), is the enzyme responsible for producing IL-1β by converting pro-IL-1β into its active form, which in turn exerts its effects via the Type 1 receptor (IL-1R1). Our previous work has indicated that caspase-1 is activated throughout the progression of diabetic retinopathy in STZ and galactosemic mice (Mohr et al., 2012; Vincent and Mohr, 2007; Trueblood et al., 2011). Caspase-1 activity was also elevated in retinal tissue samples from diabetic donors (Mohr et al., 2012; Tang et al., 2003). Müller cells are one of the retinal cell types identified as being a major source of active caspase-1 and IL-1β production (Mohr et al., 2012; Vincent and Mohr, 2007; Mohr and Vincent, 2005).

Typically, caspase-1 activation occurs in response to a bacterial infection. This mechanism is well established and usually involves sensing of the pathogen via the Nlrp3 inflammasome (Gross et al., 2011). In the diabetic retina, caspase-1 activation is unique in that it occurs in response to elevated glucose levels rather than a pathogen. Very little is known about caspase-1 activation in so called “sterile inflammation” environment. Alternative pathways that are dependent as well as independent of the Nlrp3 inflammasome have been suggested to activate caspase-1 in pathogen free conditions. For example, Receptor Interacting Protein 2 (RIP2) can act as a potential activator of caspase-1 in sterile inflammation (Humke et al., 2000; Thome et al., 1998; McCarthy et al., 1998; Gong et al., 2006; Lee et al., 2001). In Huntington’s disease, which is considered to be a sterile inflammatory disease, RIP2-mediated caspase-1 activation leads to chronic tissue inflammation and cell death (Wang et al., 2005). To date, the mechanism by which caspase-1 activity is initiated, and more importantly sustained, in the retina and retinal cells under hyperglycemic conditions is not known.

Therefore, this study aimed to identify (Stitt et al., 2013) how caspase-1 activity is induced and sustained in hyperglycemic conditions *in vivo* and *in vitro* since this process seemingly contributes to chronic inflammation seen in diabetic retinopathy and (Abcouwer, 2012) whether RIP2 acts a potential regulator of chronic caspase-1 activation.

## 2 Materials and methods

### 2.1 Antibodies and reagents

GHb kits were obtained from Glyc-Affin (Rockford, IL). TRIzol, 7-amino-4-trifluoro-methylcoumarin (AFC) was from Sigma (St. Louis, MO). Caspase-1 substrate, YVAD-fmk were from Calbiochem (San Diego, CA). Human IL-1β, IL-1 receptor antagonist (IL-1ra), and high sensitivity IL-1β ELISA was from R&D Systems (Minneapolis, MN). Rabbit polyclonal anti-RIP2 antibody (ab8428) was from Abcam (Cambridge, MA). 4%–20% Gradient Tris-SDS-PAGE gels were from BioRad (Hercules, CA). ON-TARGET*plus* SMARTpool Human RIPK2 and ON-TARGET*plus* Control siRNA Non-Targeting siRNA #1 were purchased from Thermo Scientific Dharmacon. Amaxa Cell Line Nucleofector Kit L was purchased from Lonza (Basel, Switzerland).

### 2.2 Animals

Treatment of animals conforms to the Association for Research in Vision and Ophthalmology Resolution on Treatment of Animals in Research and was approved by the University’s Animal Care and Use Committee. IL-1 receptor knockout mice (IL-1R1^−/−^) (Jackson Laboratories; strain name: *B6.129S7-Il1r1* ^*tm1jm*^) (C57BL/6 background) mice were bred using homozygous breeding pairs. At 8–10 weeks, male mice (wild-type (WT) C57BL/6 and IL-1R1^−/−^) weighing 20 g were randomly assigned to be either diabetic or non-diabetic controls. Streptozotocin (STZ) injections (60 mg/kg body wt i.p. on 5 consecutive days) were utilized to induce diabetes. Diabetes was assessed by measuring fasted blood glucose levels 2 weeks after induction of diabetes. Animals with fasted blood glucose levels >250 mg/dL were considered diabetic as previously described (Mohr et al., 2012). Diabetic animals were maintained with insulin injections (0.1–0.2 units of NPH insulin subcutaneously) as needed. Animals had free access to food and water and were maintained under a 12 h on/12 h off light cycle. GHb (glycated hemoglobin) levels were measured at the end of each study to determine severity of diabetes (Supplementary Table S1).

### 2.3 Tissue culture

#### 2.3.1 Human retinal Müller cells (hMC)

Handling of human tissue conformed to the tenets of Declaration of Helsinki for research involving human tissue. This study used donor tissue obtained from Eversight. The Institutional Review Board (IRB) of Michigan State University did not require the study to be reviewed or approved by the IRB board due to exempt status of de-identified discarded human tissue. Human Müller cells were isolated from retinal tissue of healthy donors with no history of diabetes and chronic inflammatory diseases as previously described (Kusner et al., 2004; Yego et al., 2009).

#### 2.3.2 Treatment protocols

hMC (1 × 10^6^) were treated with either 5 mmol/L glucose DMEM or 25 mmol/L glucose DMEM supplemented with 2% FBS, 1% P/S for either 48 or 96 h. Cells treated with 5 mmol/L glucose medium served as controls. For IL-1β studies, recombinant human IL-1β (1–5 ng/mL) was used. For IL-1ra studies, following pretreatment with 100 ng/mL IL-1ra for 1 h, hMC were incubated in 25 mmol/L glucose DMEM for 48 or 96 h, or in 5 mmol/L glucose DMEM plus recombinant human IL-1β (2 ng/mL) for 24 h. Each well represents one n and experiments were done using isolated Müller cells from at least 2–3 different donors to avoid reporting effects that are from one specific donor only and two different passages.

#### 2.3.3 siRNA Electroporation

hMCs were electroporated with either siRNA against RIP2 (50 nmol) or scramble RNA control (50 nmol) using a Nucleofector II device from Amaxa Biosystems (Cologne, Germany).

### 2.4 Preparation of cytosolic lysates

Following treatment, hMC were lysed in 100 μL of lysate buffer [CHAPS Buffer (100 mM HEPES, pH 7.5 containing 10% sucrose, 0.1% CHAPS), 1 mmol/L EDTA, 1 mmol/L PMSF and leupeptin (10 μg/mL)] as described previously (Vincent and Mohr, 2007).

### 2.5 Caspase-1 activity assay

Caspase-1 activities were measured as described previously (Mohr et al., 2012; Vincent and Mohr, 2007; Tang et al., 2003; Kusner et al., 2004; Yego et al., 2009; Yego and Mohr, 2010). Briefly, equal amounts of sample protein (15 μg) were incubated in the presence of the specific caspase-1 substrate (YVAD-AFC; 2.5 μmol/L) for 1 h at 32°C. AFC fluorescence was detected by a Tecan Spectra FluorPlus fluorescence plate reader (excitation: 400 nm, emission: 510 nm). Release of AFC by active caspase-1 was calculated against an AFC standard curve and expressed as pmol AFC/mg protein/min.

### 2.6 Cytokine assay

Supernatants (150 µL) from hMC treated with 5 mmol/L or 25 mmol/L glucose containing medium was added to pre-coated 96 well plates. IL-1β ELISA assays were performed according to the manufacturer’s directions. Levels of cytokine were normalized to mg of total protein.

### 2.7 Western blot analysis

Proteins (40 μg) were separated in 4%–20% SDS gradient gels and blotted on nitrocellulose membrane. Membranes were incubated with primary antibody against RIP2 (1:1000 dilution in PBS/0.05% Tween 20) overnight at 4°C followed by incubation with secondary antibody (1:5,000 dilution) for 1 h at RT and developed using LICOR Biosciences Odyssey Imaging System (Lincoln, NE). Membranes were re-probed for β-actin and relative densities of RIP2/β-actin were calculated.

### 2.8 Cell death assay

Following treatment, cells were suspended and 100 μL of cell suspension was mixed with 100 μL of trypan blue solution. Cell death was quantified using a hematocytometer as previously described (Coughlin et al., 2019b).

### 2.9 Statistical analysis

Non-parametric data were analyzed using Mann Whitney U or Kruskal–Wallis test followed by a Dunn’s *post hoc* analysis. Parametric data were analyzed using one-way ANOVA followed by a *post hoc* Tukey analysis. Normality of data was analyzed using Shapiro-Wilk Test with alpha = 0.05. For all data, we decided to present exact p-values (Mukaka, 2012; Amrhein et al., 2019). Data are expressed as mean ± SD. All statistical analysis was done using GraphPad Prism 10.

## 3 Results

### 3.1 Sustained caspase-1 activity in diabetic mice maintained by feedback through the IL-1R1 receptor

**FIGURE 1:**
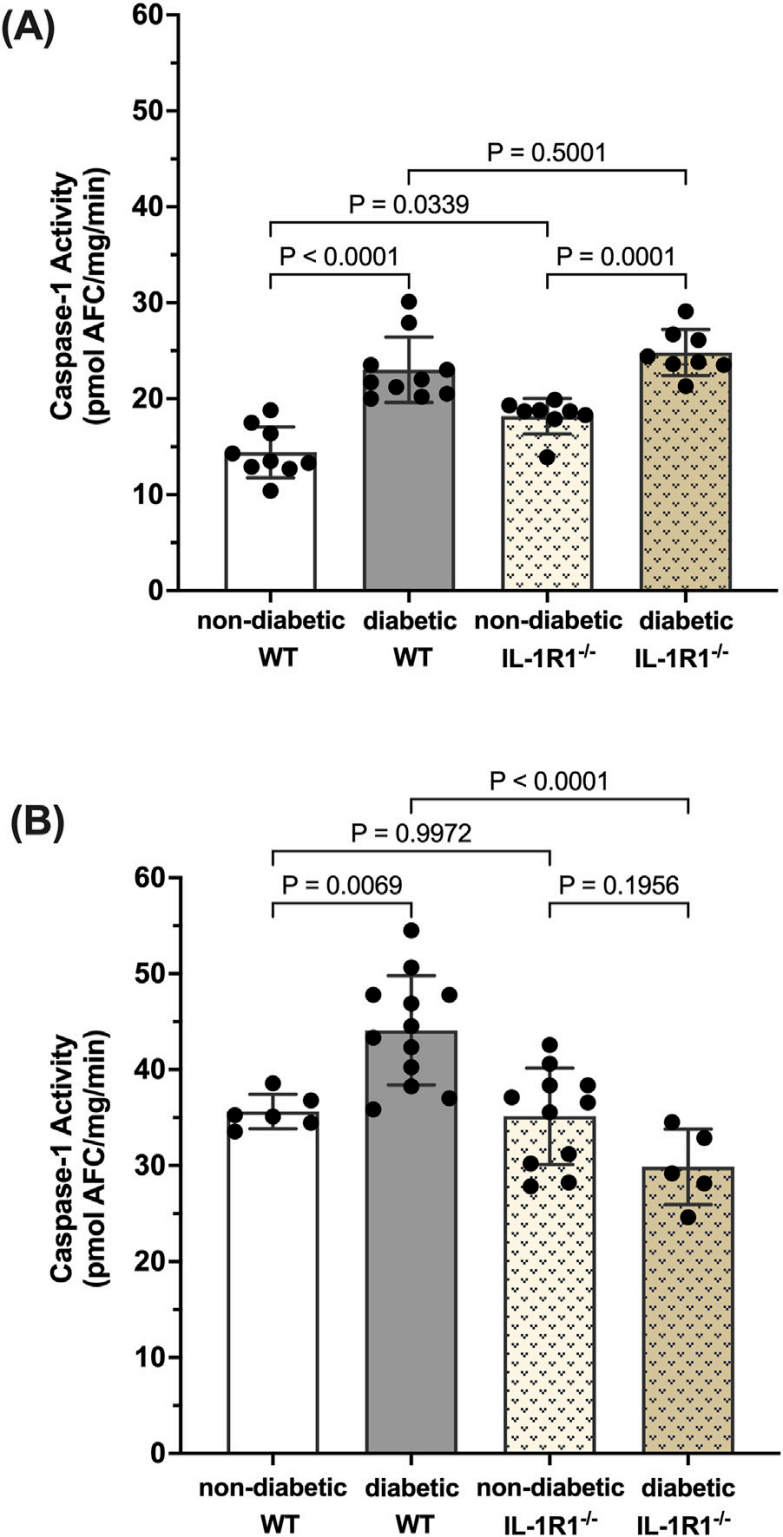
Caspase-1 activity pattern in retinas of non-diabetic and diabetic wild type and IL-1R1^−/−^mice. Retinas of non-diabetic (n = 9) and diabetic (n = 10) wild type (WT) and non-diabetic (n = 8) and diabetic (n = 8) IL-1R1^−/−^ mice were isolated at 10 weeks **(A)** and 20 weeks **(B)**. Caspase-1 activity was measured and expressed as mean ± SD (ANOVA followed by Tukey Test).

Caspase-1 activation leads to IL-1β production which signals through the IL-1R1 receptor. We have previously shown that diabetes leads to the activation of caspase-1 and IL-1β production and that blocking downstream caspase-1/IL-1β/IL-1R1 signaling using IL-1R1 knockout mice prevented the formation of acellular capillaries and Müller cell death (Vincent and Mohr, 2007; Mohr and Vincent, 2005). Thus, we were interested to understand how caspase-1 activity is regulated in such a mouse model. After 10 weeks of diabetes, caspase-1 activity was significantly increased in both diabetic WT and diabetic IL-1R1^−/−^ mice by 59.6% ± 15.3% and 33.2% ± 9.8% respectively compared to non-diabetic mice (Figure 1A). At 20 weeks of diabetes, caspase-1 activity was increased by 30.2% ± 3.8% in the retinas of diabetic WT mice compared to non-diabetic WT mice. However, there was no significant increase in caspase-1 activity in retinas of diabetic IL-1R1^−/−^ mice compared to non-diabetic IL-1R1^−/−^ or WT mice indicating that caspase-1 activation progresses from an IL-1R1 independent mechanism at 10 weeks of diabetes to an IL-1R1 dependent mechanism at 20 weeks of diabetes (Figure 1B). The overall increase of caspase-1 activity levels at 20 weeks of diabetes compared to 10 weeks of diabetes (p = 0.004) was slightly due to increased background fluorescence that excited and emitted at the same wavelength as the caspase-1 substrate and mostly due to aging. Diabetes is considered accelerated aging (Shapiro et al., 2023; Bahour et al., 2021; Antal et al., 2022) and aging is promoted by increased inflammatory events such as the activation of caspase-1 (Baechle et al., 2023; Ferrucci and Fabbri, 2018; Li et al., 2023). The interesting observation in regard to aging needs to be studied and further explored but went beyond the scope of this manuscript. The most important observation for this study, the shift from IL-1R1 independent to IL-1R1 dependent mechanism of diabetes-induced caspase-1 activation strongly suggests a caspase-1/IL-1β/IL-1R1 feedback signaling mechanism that seems to be responsible for chronic IL-1-mediated inflammation in the diabetic retina.

### 3.2 Hyperglycemia-induced caspase-1/IL-1β/IL-1R1 feedback signaling in human Müller cells

**FIGURE 2:**
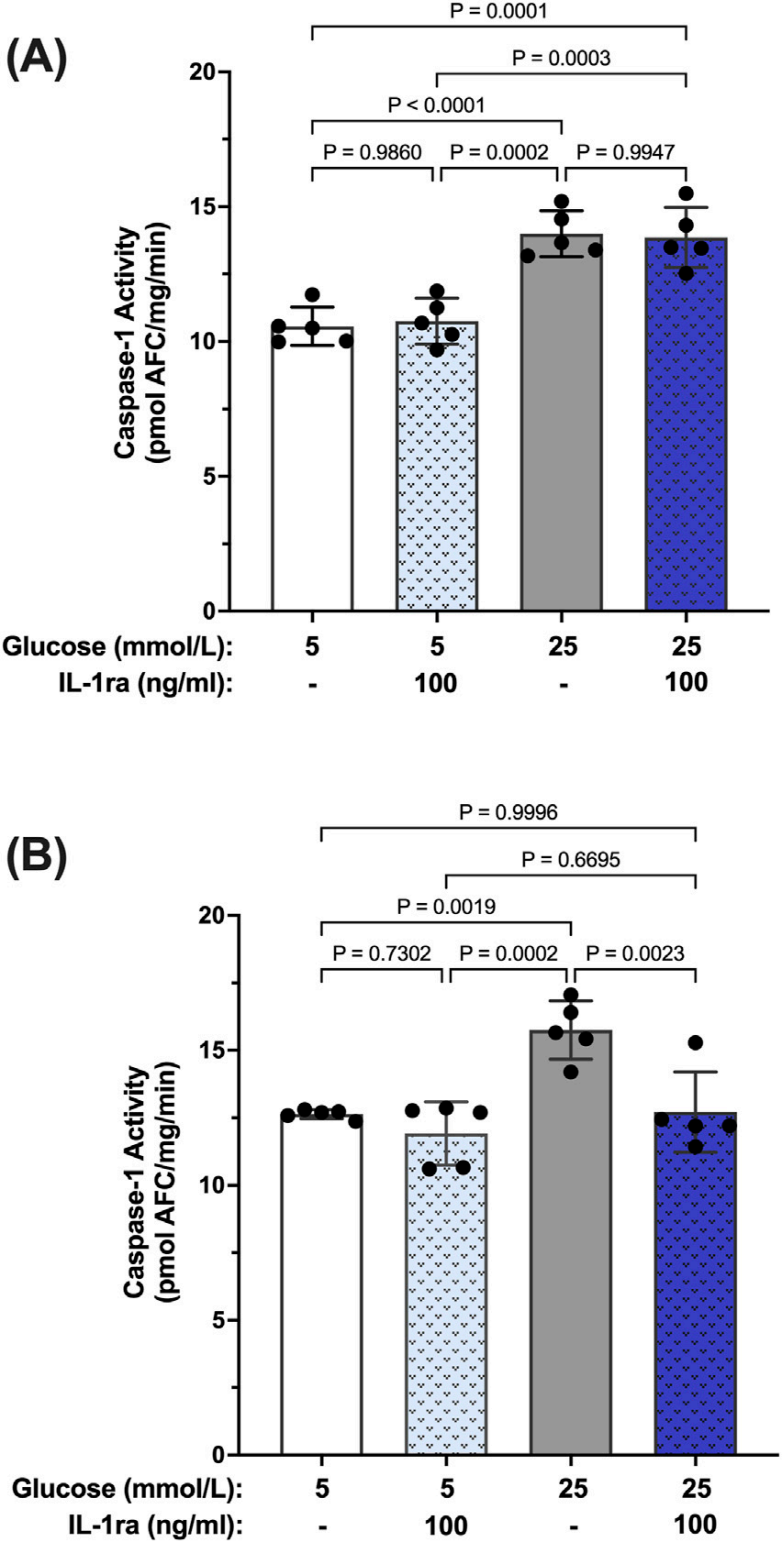
Inhibition of hyperglycemia-induced caspase-1 activity in human Müller cells by IL-1 receptor antagonist. hMCs were cultured in either 5 mmol/L or 25 mmol/L glucose media in the presence or absence of 100 ng/mL IL-1ra for **(A)** 48 h (n = 5) or **(B)** 96 h (n = 5). Caspase-1 activity was measured and expressed as mean ± SD (ANOVA followed by Tukey Test).

To further establish a hyperglycemia-induced caspase-1/IL-1β/IL-1R1 feedback loop we used isolated human Müller cells (hMC). Previously we have shown that hyperglycemia induces caspase-1 activation and IL-1β production in hMCs (Mohr et al., 2012; Vincent and Mohr, 2007; Yego et al., 2009). To determine whether caspase-1 activation progresses from an IL-1R1 independent mechanism to an IL-1R1 dependent mechanism in this cellular model, hMCs were treated with 5 mmol/L or 25 mmol/L glucose media in the presence or absence of an IL-1 receptor (IL-1ra) antagonist for 48 or 96 h. At 48 h, caspase-1 activity was increased by 32.5% ± 3.6% in hyperglycemic conditions compared to normal. IL-1ra treatment had no significant effect on caspase-1 activity (Figure 2A). At 96 h, treatment with IL-1ra led to a 97.7% ± 5.3% reduction in caspase-1 activity in cells incubated in hyperglycemic conditions indicative of a caspase-1/IL-1β/IL-1R1 feedback signaling (Figure 2B).

**FIGURE 3:**
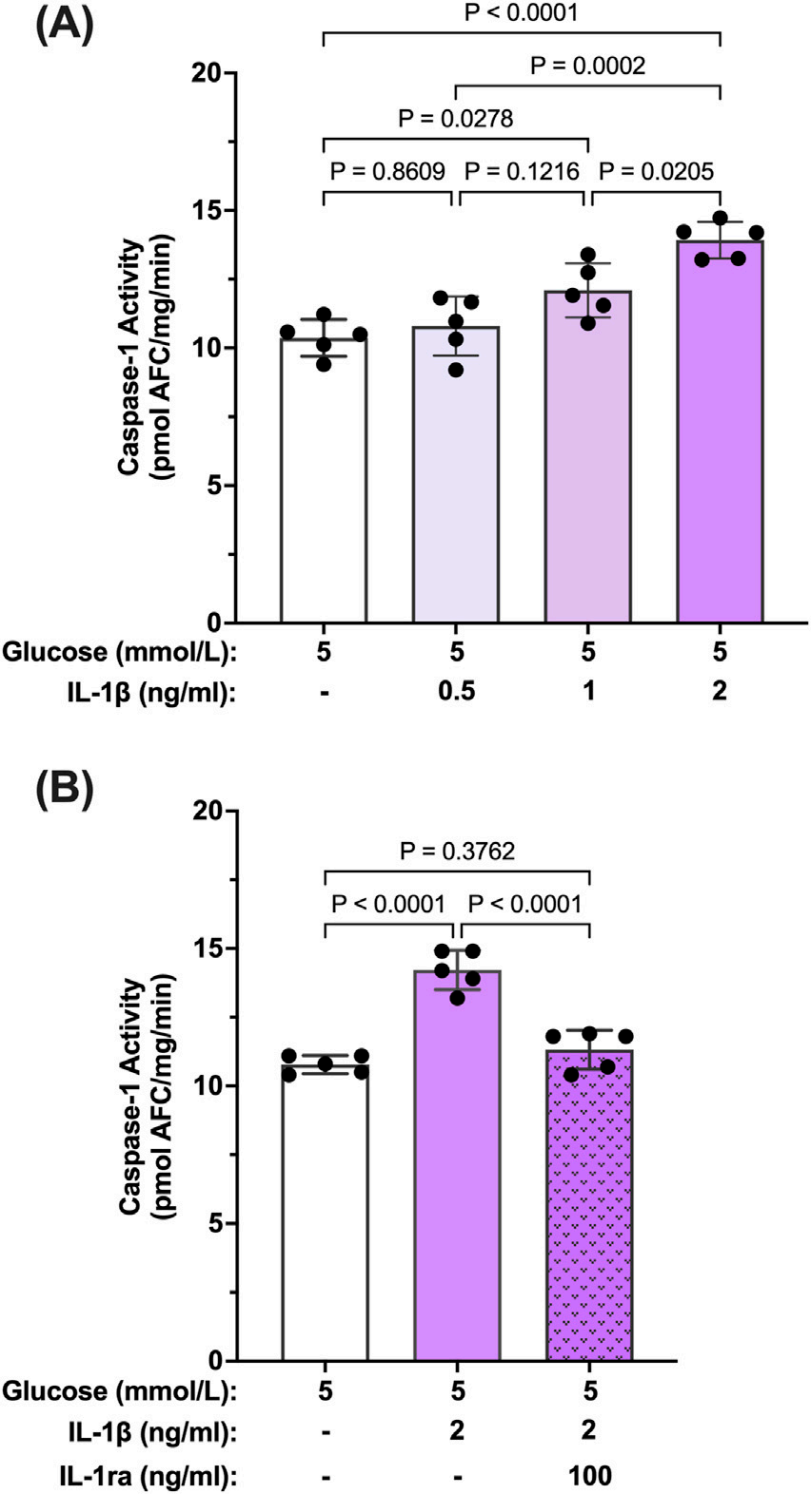
Inhibition IL-β-induced caspase-1 activity in human Müller cells by IL-1 receptor antagonist. **(A)** hMCs were treated in 5 mmol/L glucose or 5 mmol/L glucose + IL-1β (0.5, 1, or 2 ng/mL; n = 5) containing media for 24 h **(B)** hMCs were treated with IL-1ra (100 ng/mL) in either 5 mmol/L glucose or 5 mmol/L glucose + IL-1β (2 ng/mL) media for 24 h (n = 5). Caspase-1 activity was measured and expressed as mean ± SD (ANOVA followed by Tukey Test).

Reports in the literature have suggested that IL-β itself can promote activation of caspase-1 via signaling through the IL-1R1 receptor (Dinarello et al., 1950; Hoffman et al., 2004). Thus, we tested whether IL-1β can induce caspase-1 activity in hMCs. hMCs were treated with increasing concentrations of IL-1β (0.5–2 ng/mL) in 5 mmol/L glucose conditions for 24 h. IL-1β induced caspase-1 activation in a concentration dependent fashion demonstrating that IL-1β is capable of inducing caspase-1 activity (Figure 3A). Treatment with IL-1ra led to an 87.2% ± 3.4% inhibition of IL-1β-induced caspase-1 activity demonstrating that the effects seen by IL-1β were mediated by the IL-1 receptor (Figure 3B). These data demonstrate that hyperglycemia induced sustained caspase-1 activity in hMCs via caspase-1/IL-1β/IL-1R1 feedback signaling comparable to what we observed in the animal model.

### 3.3 RIP2 mediated caspase-1 activation by hyperglycemia and IL-1β

**FIGURE 4:**
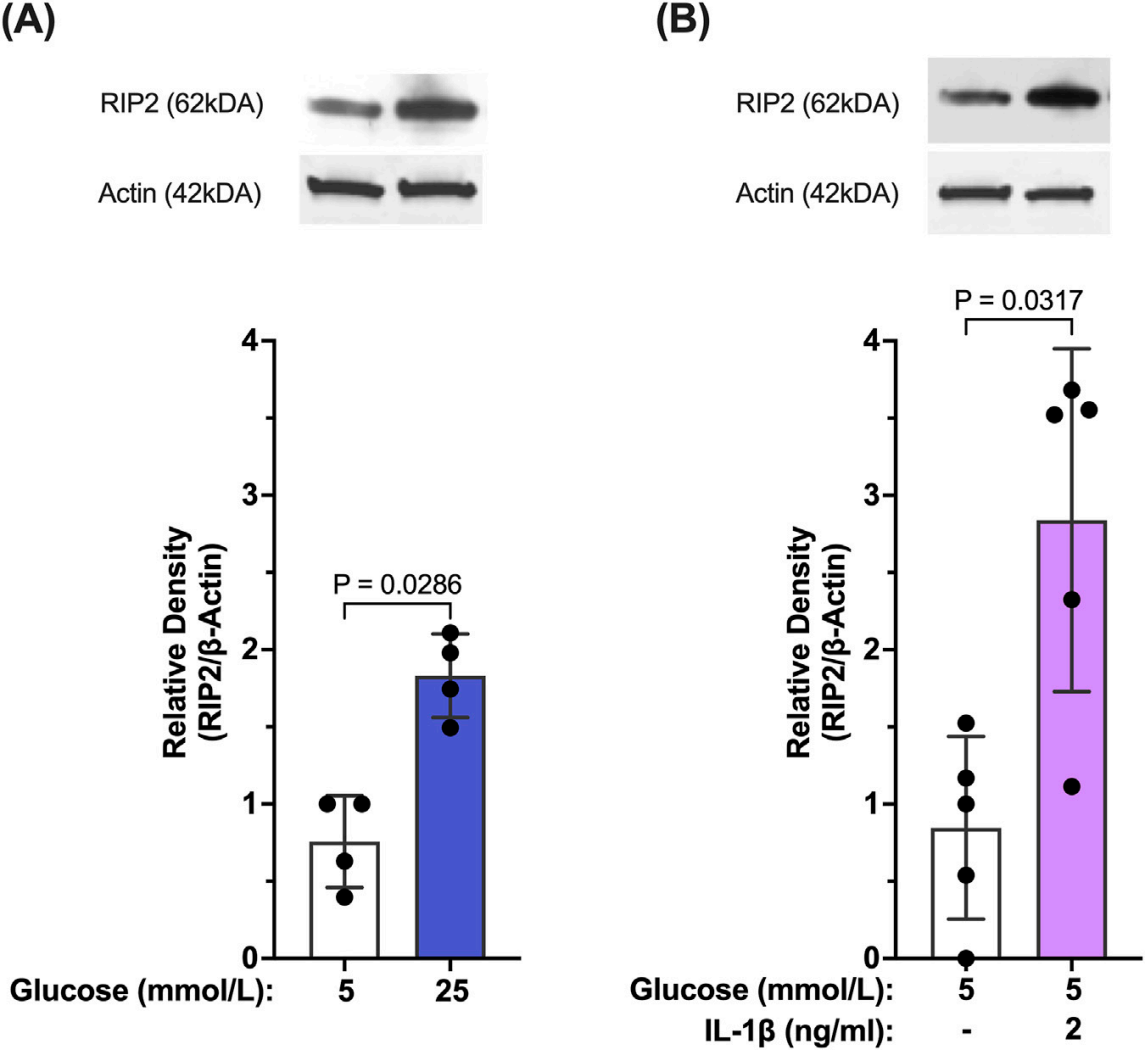
Hyperglycemia- and IL-β- mediated upregulation of RIP2 in Müller cells. **(A)** hMCs were incubated in either 5 mmol/L glucose or 25 mmol/L glucose media for 48 h. RIP2 protein levels were determined by Western blot analysis, normalized to β-actin, and expressed as mean ± SD (n = 4). **(B)** hMCs were incubated in either 5 mmol/L glucose or 5 mmol/L glucose + IL-1β (2 ng/mL) for 24 h. RIP2 protein levels were determined by Western blot analysis, normalized to β-actin, and expressed as mean ± SD (n = 5) (Kruskal Wallis followed by Dunn’s test).

Our results so far indicate that caspase-1 activation in Müller cells is seemingly maintained by two stimuli. Initially, activation of caspase-1 is predominantly driven by hyperglycemia. Once activated, caspase-1 activity is sustained by feedback signaling of IL-1β through the IL-1 receptor. We were interested whether both stimuli activate caspase-1 by the same mechanism. Reports have shown that in “sterile inflammation” RIP2 played a significant role in hyperglycemia-induced caspase-1 activation, thus, we tested whether both stimuli upregulate RIP2. As shown in Figure 4A, RIP2 protein is significantly upregulated in hMCs cultured in 25 mmol/L glucose (77.6% ± 25.5%) compared to control. Since RIP2 upregulation was crucial for hyperglycemia-driven caspase-1 activation, we further investigated the role of RIP2 in IL-1β-induced caspase-1 activation. Treatment of hMCs with IL-1β in 5 mmol/L glucose media led to a 2.38 ± 0.6 fold increase in RIP2 protein levels (Figure 4B) demonstrating that both stimuli, hyperglycemia and IL-1β, upregulate the expression of RIP2.

### 3.4 Inhibition of hyperglycemia- and IL-β-induced caspase-1 activity by RIP2 knockdown

**FIGURE 5:**
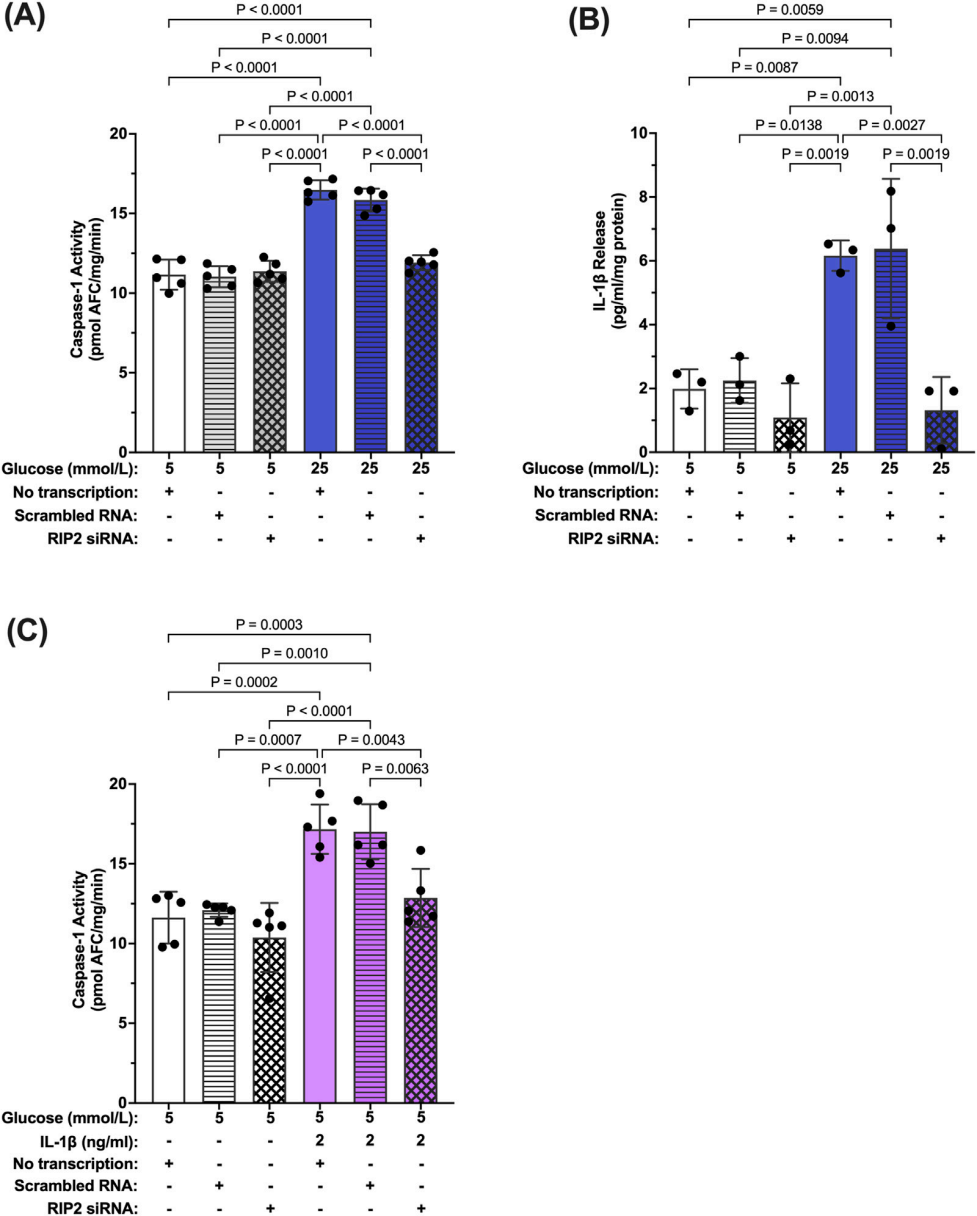
Inhibition of hyperglycemia- and IL-1β-induced caspase-1 activity by RIP2 knockdown. **(A)** hMCs transfected with either scramble RNA or siRNA were incubated in 5 mmol/L glucose or 25 mmol/L glucose media for 48 h. Caspase-1 activity was assessed and expressed as mean ± SD (n = 5). hMCs without transfection served as controls. **(B)** IL-1β release was measured using ELISA assays. IL-1β levels are expressed as mean ± SD (n = 3). **(C)** hMCs transfected with either scramble RNA or siRNA were incubated in 5 mmol/L glucose or 5 mmol/L glucose + IL-1β (2 ng/mL) for 24 h. Caspase-1 activity was assessed and expressed as mean ± SD (n = 5) (ANOVA followed by Tukey Test–only significant *p* values are presented).

To confirm that increased RIP2 levels are indeed responsible for increased hyperglycemia-induced caspase-1 activity, siRNA against RIP2 was used (Supplementary Figure S2). Caspase-1 activity was significantly increased in hMCs transfected with scramble siRNA or left non-transfected in hyperglycemic conditions compared to control (no transfection). Knockdown of RIP2 attenuated high glucose-induced caspase-1 activity by 85.8% ± 1.8% demonstrating that RIP2 is necessary for hyperglycemia-induced caspase-1 activity (Figure 5A). Consistently, knockdown of RIP2 also prevented IL-1β production under these conditions (Figure 5B). siRNA against RIP2 also prevented IL-1β-induced caspase-1 activation as seen in Figure 5C. RIP2 is not only involved in hyperglycemia-induced caspase-1 activation but also promotes caspase-1 activation by IL-1β. Together, this indicates that RIP2 is an important mediator of caspase-1/IL-1β/IL-1R1 feedback signaling that controls Müller cell inflammatory responses.

### 3.5 Hyperglycemia-induced Müller cell death by caspase-1/IL-1β/IL-1R1 feedback signaling

**FIGURE 6:**
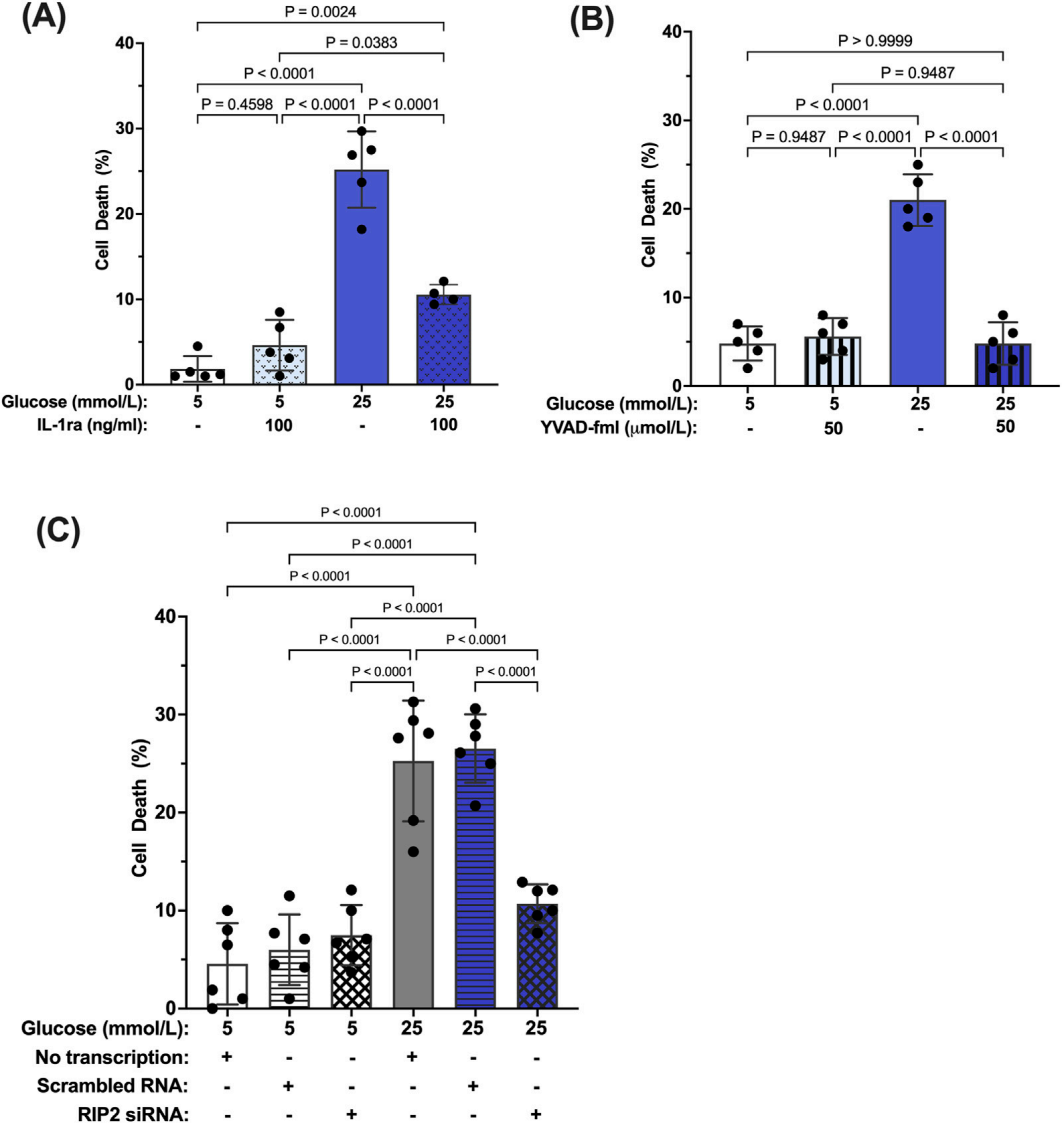
Inhibition of hyperglycemia-induced Müller cell death by IL-1ra, YVAD-fmk, and RIP2 siRNA. hMCs were cultured in either 5 mmol/L or 25 mmol/L glucose media in the presence or absence of **(A)** 100 ng/mL IL-1ra (n = 5), **(B)** 50 μmol/L YVAD-fmk (n = 5), or **(C)** RIP2 siRNA (n = 6) for 96 h. At 96 h, cell death was determined using the Trypan Blue Exclusion assay and expressed as mean ± SD (Kruskal Wallis followed by Dunn’s test).

We have previously shown that prolonged exposure to hyperglycemia causes Müller cell death (Mohr and Vincent, 2005; Yego et al., 2009). To determine the role of caspase-1/IL-1β/IL-1R1 feedback signaling in Müller cell death, hMCs were treated with 5 mmol/L or 25 mmol/L glucose media in the presence or absence of IL-1ra, the caspase-1 specific inhibitor YVAD-fmk, or RIP2 siRNA for 96 h. IL-1ra treatment prevented hyperglycemia-induced cell death demonstrating that feedback signaling affects proper Müller cell function and viability (Figure 6A). In addition, upstream inhibition of the caspase-1/IL-1β signaling using YVAD-fmk or RIP2 knock down also prevented hyperglycemia mediated cell death of Müller cells (Figures 6B,C). These data indicate that interference in caspase-1/IL-1β/IL-1R1 feedback signaling at any point of the pathway is beneficial for Müller cell survival.

## 4 Discussion

**FIGURE 7:**
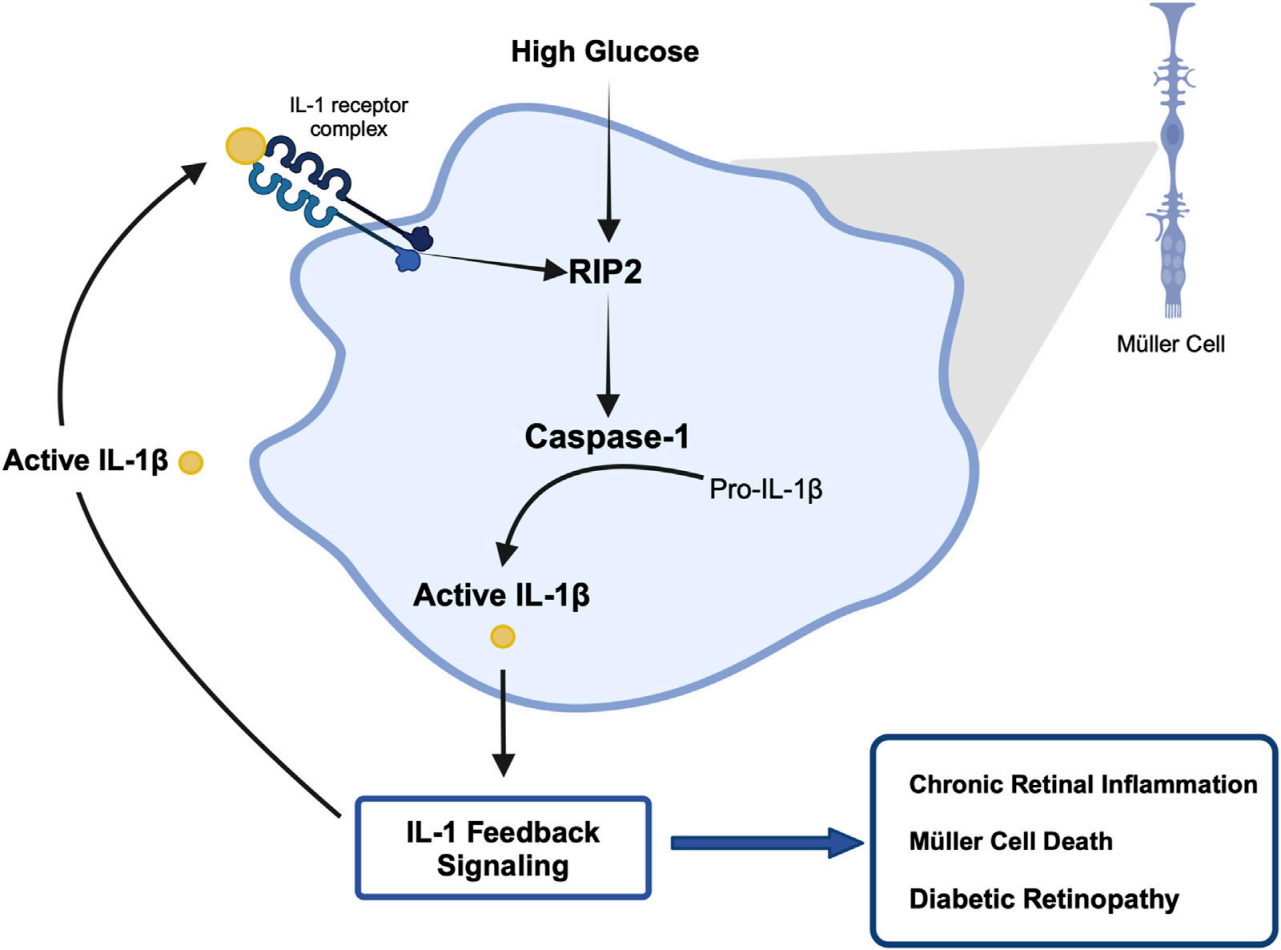
Proposed mechanism of hyperglycemia-induced chronic caspase-1 activity.

The importance of inflammation in the progression of diabetic retinopathy has become increasingly apparent. A variety of pro-inflammatory cytokines have been identified in the vitreous of patients compared to healthy individuals, among them IL-1β (Yoshida et al., 2004; Sfikakis et al., 2005; Demircan et al., 2006; Joussen et al., 2009; Li et al., 2009), (Bromberg-White et al., 2013). Our previous studies have shown that caspase-1 activity is consistently increased in the retinas of diabetic animals and patients (Mohr et al., 2012; Vincent and Mohr, 2007). This new study provides novel mechanistic insights into the process of chronic caspase-1 activation and IL-β production in diabetic retinopathy. Using the IL-1R1^−/−^ mice we identified that diabetes-induced caspase-1 activity progresses from an IL-1R1 independent mechanism to an IL-1R1 dependent mechanism throughout disease progression. This is consistent with our previous observation that knock down of the IL-1R1 receptor prevented the development of diabetic retinopathy (Vincent and Mohr, 2007; Feenstra et al., 2013). These data provide for the first time an indication of a caspase-1/IL-1β/IL-1R1 feedback signaling mechanism that keeps caspase-1 active in the diabetic retina. Using Müller cells known to produce active caspase-1 and IL-1β under hyperglycemic conditions we confirmed that prolonged exposure to hyperglycemia leads to caspase-1/IL-1β/IL-1R1 feedback signaling causing sustained caspase-1 activity. We were able to show that hyperglycemia initiates caspase-1 activity and IL-1β continues to promote caspase-1 activation. In addition, activation of caspase-1 by both stimuli, hyperglycemia and IL-1β, was mediated by RIP2 as determined by RIP2 knockdown experiments. Finally, we determined that activation of the caspase-1/IL-1β/IL-1R1 feedback is detrimental leading to Müller cells death. Hyperglycemia is not only detrimental for Müller cells *in vitro* but most importantly *in vivo* as previously shown (Feenstra et al., 2013; Coughlin et al., 2017). Taken together, this indicates that hyperglycemia induces caspase-1 activation and IL-1β production initiating a seemingly IL-1β driven caspase-1/IL-1β/IL-1R1 feedback cycle that is detrimental to the viability of retinal cells such as Müller cells and promotes the development and progression of diabetic retinopathy (Figure 7).

The phenomenon of a caspase-1/IL-1β/IL-1R1 feedback signaling is intriguing for several reasons. Although our study used Müller cells to demonstrate hyperglycemia-induced caspase-1/IL-1β/IL-1R1 feedback signaling, other retinal cells might be capable of producing similar feedback cycles leading to sustained caspase-1 activation and prolonged IL-1β production. Several retinal cell types such as astrocytes and microglia have been identified too to produce IL-1β when exposed to elevated glucose levels (Liu et al., 2012; Krady et al., 2005; Liu et al., 2009). This leaves the strong possibility that IL-1β produced by one retinal cell type feeds into IL-1β feedback signaling of another retinal cells type even further augmenting IL-1β production. In the beginning IL-1β production will be low since there are several effective mechanisms of IL-1 degradation (Ito et al., 1996; Evavold and Kagan, 2022; Vijayaraj et al., 2021) but once levels of IL-1β exceed those defense mechanisms it will become detrimental. Despite our *in vitro* studies showing that autocrine activation is sufficient to induce caspase-1/IL-1β/IL-1R1 feedback signaling the combination of several IL-1β-producing cells amplifying feedback signaling might be necessary to reach high enough IL-1β levels for IL-1β to drive caspase-1 activation via the IL-1R1 in the diabetic retina. This could explain the long duration of diabetes required for caspase-1 activity to become dependent on IL-1β feedback *in vivo*. However, more studies need to be done to positively confirm this concept of caspase-1/IL-1β/IL-1R1 feedback signaling in other retinal cell types. Future studies will also have to identify how other cytokines such as TNFα that have been associated with the development of diabetic retinopathy influence the regulation of the caspase-1/IL-β/IL-1R1 feedback signaling.

Looking deeper into the switch of caspase-1 activation from IL-1R1 independent to IL-1R1 dependent mechanisms despite the presence of an obvious hyperglycemic environment. Müller cells are unique in their metabolism since they derive their energy primarily from glycolysis and are known to take up large amounts of glucose (Winkler et al., 2000). Although hyperglycemia initially drives caspase-1 activation, over time glucose consumption surprisingly does not seem to serve as mediator of prolonged caspase-1 activity. More detrimental seems that fact that cells are unable to compensate for the increased levels of IL-1β, which itself is capable of driving caspase-1 activation. This leads to caspase-1 activation being driven solely by IL-1β feedback signaling, which explains the effectiveness of IL-1ra treatment since glucose is playing a minor role in caspase-1 activation once the feedback signaling is set in motion. How much glucose is needed to trigger caspase-1 activation and to support ongoing caspase-1/IL-1β/IL-1R1 feedback signaling has to be determined in more detail in future studies. Interestingly, although caspase-1 activation during this feedback signaling is mediated by two distinct stimuli, hyperglycemia and IL-1β, both pathways are controlled by one regulator, RIP2. RIP2, a 62 kDa CARD domain containing protein, can act as a scaffold protein capable of binding and activating pro-caspase-1 via CARD-CARD interaction (Humke et al., 2000; Thome et al., 1998; Wang et al., 2005). Aberrant RIP2 activity has been implicated as a driver of inflammation in diseases such as Huntington’s Disease, where it causes increased caspase-1 activation and IL-1β production, ultimately leading to neuronal cell death (Wang et al., 2005). For the first time this study shows that RIP2 plays a role in hyperglycemia-mediated caspase-1 activation and cell death in retinal Müller cells. This is interesting since the more prominent mechanisms of caspase-1 activation seem to be inflammasome-mediated mechanisms involving NLRP3 which has also been linked to RIP2 (Gross et al., 2011), (Lim et al., 2020). NLRP3 upregulation has been shown in some retinal cells (Devi et al., 2012) but was not observed in our studies using human Müller cells (data not shown).

Prolonged activation of caspase-1 and IL-1β production causes Müller cell death *in vitro* and *in vivo* (Tang et al., 2003; Mohr and Vincent, 2005). Müller cell death in diabetic retinopathy was first identified in 1980, however, few studies have looked at actual mechanisms underlying Müller cell death (Vincent and Mohr, 2007; Yego et al., 2009; Yego and Mohr, 2010; Feenstra et al., 2013; Hori and Mukai, 1980). Intervention in the caspase-1/IL-1β/IL-1R1 feedback signaling by inhibition of caspase-1 as shown in this study or by the IL-1 receptor as we previously reported prevents Müller cell death in diabetic retinopathy demonstrating the importance of this signaling pathway to Müller cell viability (Feenstra et al., 2013). The protective effect of caspase-1 inhibition on Müller cell viability is not surprising. Increasing evidence shows that there are a number of modes of programmed cell death in addition to the classical apoptosis (Kroemer et al., 2009; Galluzzi et al., 2012). One of the more recently identified types of cell death, termed pyroptosis, is an inherently inflammatory based cell death defined as being caspase-1 dependent and preventable by inhibition of caspase-1 (Galluzzi et al., 2012; Yu et al., 2021). This current study demonstrates that hyperglycemia-induced Müller cell death fulfills all criteria for pyroptosis and links the pro-inflammatory function of caspase-1 and IL-1β production to cell death. Excessive IL-1β production by Müller cells might not only affect viability of Müller cells but also affect viability of surrounding retinal cells as we have previously shown for endothelial cells (Busik et al., 2008). Furthermore, Müller cell death preceded acellular capillary formation suggesting a potential role for inflamed Müller cells and Müller cell death in the progression of diabetic retinopathy.

In this study we have outlined a mechanism of prolonged caspase-1 activation that contributes to chronic inflammatory events in the diabetic retina and while set in motion by hyperglycemia seemingly becomes independent of its original hyperglycemic insult. If confirmed in diabetic patients, it could potentially explain why diabetic retinopathy still progresses despite good control of blood glucose levels. It also might open new venues for treatment of diabetic retinopathy. Currently the most common therapy seeks to inhibit VEGF-A (vascular endothelial cell growth factor-A), a growth factor that promotes increased vascular permeability, macular edema, and neovascularization (Salam et al., 2011; Al-Latayfeh et al., 2012; Bandello et al., 2012; Tremolada et al., 2012; Gupta et al., 2013). While these drugs have been successful in some patients, they do not provide reliable benefits for all patients. Our data suggest that treatments against inflammatory events are potentially more beneficial than inhibition of growth factors such as VEGF-A since several studies have shown that IL-1β promotes increased production of VEGF-A (Tanaka et al., 2000; Solà-Villà et al., 2006; Jung et al., 2001). Although there are a couple of drugs on the market that target the caspase-1/IL-1β complex none of them have been tested in the context of diabetic retinopathy. Inhibition of caspase-1/IL-1β/IL-1R1 feedback signaling may provide new therapeutic options upstream of VEGF, without inhibiting VEGF actions directly which are needed for neuroprotection (Coughlin et al., 2019b).

## Data Availability

The raw data supporting the conclusions of this article will be made available by the authors, without undue reservation.
