# Supplementary material for: Interleukin-1 receptor-dependent and -independent caspase-1 activity in retinal cells mediated by receptor interacting protein 2
Source: Front Cell Dev Biol. 2024 Oct 16;12:1467799. doi: 10.3389/fcell.2024.1467799 (PMC11525982; doi:10.3389/fcell.2024.1467799)
Supplement: Supplementary file 1 [file Table1.DOCX]

Supplementary Material

# Supplementary Figures and Tables

## Supplementary Table 1:

| **Experimental Group** | **Duration of Diabetes (weeks)** | **GHb (%)** | **GHb (nmol/L)** |
| --- | --- | --- | --- |
| Normal WT | 10 | 4.3 ± 0.6 | 24 ± 6 |
| Diabetic WT | 10 | 10.5 ± 4.0 | 91 ± 39 |
| Normal IL-1R1-/- | 10 | 3.4 ± 0.4 | 13 ± 4 |
| Diabetic IL-1R1-/- | 10 | 10.4 ± 0.9 | 90 ± 9 |
| Normal WT | 20 | 4.4 ± 0.8 | 24 ± 8 |
| Diabetic WT | 20 | 13.2 ± 0.8 | 121 ± 8 |
| Normal IL-1R1-/- | 20 | 3.7 ± 0.2 | 17 ± 2 |
| Diabetic IL-1R1-/- | 20 | 9.4 1.1 | 79 ± 11 |

**Characteristics of Experimental Groups.** GHb (glycated hemoglobin) levels were measured at the end of each study to determine severity of diabetes and are presented as mean ± SD.

## Supplementary Figure 2:

**Inhibition of hyperglycemia-induced upregulation of RIP2 by RIP2 siRNA.** hMCs transfected with either scramble RNA or siRNA were incubated in 5 mmol/L glucose or 25 mmol/L glucose media for 48 hours. hMCs without transfection served as controls. RIP2 protein levels were determined by Western Blot analysis. β-actin served as control.
